# Draft Genome Sequence of *Bacillus subtilis* subsp. *natto* Strain CGMCC 2108, a High Producer of Poly-γ-Glutamic Acid

**DOI:** 10.1128/genomeA.00426-16

**Published:** 2016-05-26

**Authors:** Siyuan Tan, Yonghong Meng, Anping Su, Chen Zhang, Yuanyuan Ren

**Affiliations:** College of Food Engineering and Nutritional Science, Shaanxi Normal University, Shaanxi, China

## Abstract

Here, we report the 4.1-Mb draft genome sequence of *Bacillus subtilis* subsp. *natto* strain CGMCC 2108, a high producer of poly-γ-glutamic acid (γ-PGA). This sequence will provide further help for the biosynthesis of γ-PGA and will greatly facilitate research efforts in metabolic engineering of *B. subtilis* subsp. *natto* strain CGMCC 2108.

## GENOME ANNOUNCEMENT

*Bacillus subtilis* subsp. *natto*, a spore-forming, aerobic, gram-positive bacterium is thought to be a potential host strain used in the production of the traditional Japanese food “natto” made from soybeans and is a potent producer of poly-γ-glutamic acid (γ-PGA) (1). As a consequence, there has been growing interest in the biotechnological applications of this host strain due to both established biological information and intriguing physiological characteristics. Recently, efforts have further expanded the genetic toolbox for *B. subtilis* subsp. *natto* and isolated strains that are high-level producers of γ-PGA.

Although type strain BEST195 DNA has been available for some time (1), it is not the highest producer of γ-PGA. *B. subtilis* subsp. *natto* CGMCC 2018 has been found to be a higher producer of γ-PGA (2), and is widely used in the fields of foods, cosmetics, pharmaceuticals, and water treatment due to its outstanding qualities such as biodegradability, film-reforming property, moisture-retention property, and edibility (3–7). We studied the effect of calcium on the activity of α-ketoglutaric acid dehydrogenase (OGDH) *in vivo* and *in vitro*, as well as expression abundances of OGDH and poly-glutamate synthesizing enzyme (PGSB). As a result, we found that the activities of OGDH and PGSB are regulated by Ca2+ at the transcriptional level and, in turn, have a significant effect on γ-PGA production (8).

Samples were sent to the Beijing Genomics Institute at the High-Throughput Sequencing Facility (HTSF) for whole-genome shotgun sequencing using an Illumina HiSeq2000 sequencer (Beijing, China). The raw sequence data comprise a total of 90 reads that provide high sampling coverage of the genome (34.39-fold coverage). The reads were assembled using SOAPdenovo version 1.05 (9). This led to a genome assembly containing 35 contigs (each at a length of ≥310 bp). The gaps among the scaffolds were filled by a long PCR experiment and three large sequences were finally constructed, including *B. natto* CGMCC 2018 with 4,122,154 bp containing two plasmids, one with 5,820 bp and the other with 65,774 bp.

The draft genome of strain CGMCC 2108 was analyzed and annotated using the NCBI Prokaryotic Genome Annotation Pipeline (PGAP) (http://www.ncbi.nlm.nih.gov/genome/annotation_prok). The draft genome sequence of strain CGMCC 2108 has a G+C content of 43.26%. The genome contains 4,487 predicted open reading frames and 4,365 predicted protein-coding sequences. There are 87 tRNA genes, 10 rRNA genes, and 5 noncoding RNA (ncRNA) genes predicted from this assembly.

### Nucleotide sequence accession numbers.

This whole-genome shotgun project has been deposited at DDBJ/EMBL/Gen Bank under the accession numbers CP014471 to CP014473.
